# Dr. João Carlos Pinto Dias, master and mentor! (ê1948 †2021)

**DOI:** 10.1590/0037-8682-0076-2026

**Published:** 2026-04-17

**Authors:** Liléia Gonçalves Diotaiuti

**Affiliations:** 1 Fundação Oswaldo Cruz Minas, Instituto René Rachou, Belo Horizonte, Brazil.

*“De maneira geral, os estudos epidemiológicos sobre a doença de Chagas humana têm abordado isoladamente as diferentes expressões da interação biológica entre o Trypanosoma cruzi e o homem. Tais estudos raramente alcançam as dimensões contextuais ou históricas do fenômeno. Parcelas destas expressões se traduzem em considerável acervo de dados estatísticos e no estabelecimento de relações de causalidade e repercussão nos níveis imediatos ou mediatos da endemia. ... Sem embargo, uma perspectiva epidemiológica mais ampla tem surgido de esforços isolados, acompanhando uma tendência de vários grupos que situam o binômio saúde-doença no contexto socio-cultural. Tais esforços se motivam numa visão epistemológica do trabalho científico, buscando a função social do pesquisador através da retomada da ideia do homem como sujeito e fim maior da atividade da Ciência”* ^1^ *.*

Epidemiological studies on human Chagas disease have addressed the varying expressions of biological interactions between *Trypanosoma cruzi* and humans. Such studies have rarely explored the contextual dimensions or historical aspects of a phenomenon. Some of these expressions translate into a considerable collection of statistical data and the establishment of causal relations and repercussions at the immediate or intermediate levels of the endemic disease. However, a broader epidemiological perspective has emerged from isolated efforts, alongside a tendency among various groups to situate the health-disease binomial within the sociocultural context. These efforts are motivated by an epistemological perspective of scientific work that emphasizes the social function of the researcher, advocating for the idea of ​​humans as both the subject and the ultimate purpose of scientific activity^1^.

**Legend:**
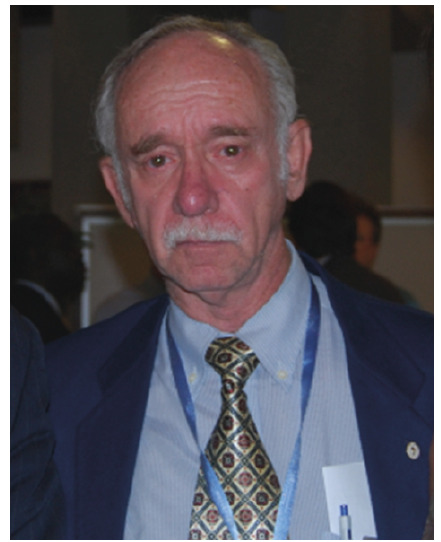
João Carlos Pinto Dias during his participation in a WHO meeting.

On the last day of 2025, we lost one of Brazil’s most brilliant minds. João Carlos Pinto Dias, son of Nicia Pinto Dias and Emmanuel Dias, was a medical doctor trained at the Faculty of Medicine of Ribeirão Preto in 1963, with a Master’s degree (1974) and a Doctorate (1982) from the Universidade Federal de Minas Gerais . He dedicated his life to the study of Chagas disease and became a national and international reference on various topics related to clinical medicine, epidemiology, and disease control. His scientific importance was contrasted by the simplicity with which he engaged with everyone, always disposed to listen and share his knowledge and enthusiasm, whether as a government minister or an agent of municipal health. While still a student in Ribeirão Preto, where he enjoyed a very stimulating and creative university environment, his commitment to social justice had already manifested itself through the student movement, reinforced by his wife and companion throughout his life.

It is impossible to summarize the achievements of João Carlos in a simple publication; therefore, I will highlight some that come to mind and inspire my admiration.

João Carlos grew up breathing Chagas disease through the activities of his father, Emmanuel, one of the most important disciples of Chagas. Emmanuel founded the Fiocruz research outpost in the small municipality of Bambui, located in the western part of Minas Gerais. This outpost was dedicated to investigating Chagas disease in the region and is now named in his honor (Posto Avançado de Pesquisas Emmanuel Dias). João Carlos was originally from the city of Rio de Janeiro; however, because of his father’s work in Bambui, the family moved to Belo Horizonte, the capital of the state of Minas Gerais^2^. João Carlos grew up between the backyard of his house on Chumbo Street, now known as Estevão Pinto Street in honor of his maternal grandfather, and the farm in Bambui, following his father around as a young boy, with an insecticide fogger strapped on his back. After the premature death of his father and mentor, he would follow in his footsteps, assuming the position of chief of the outpost (1974-1990). There, he developed as a researcher at Fiocruz and undertook numerous research projects.

**Legend:**
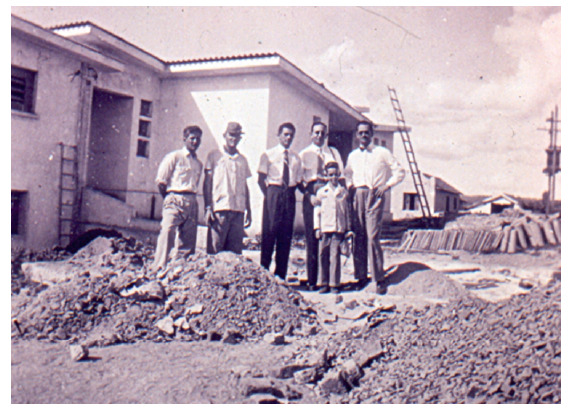
Young João Carlos accompanying his father, Dr. Emmanuel Dias, during the construction of the Posto Avançado de Pesquisa in Bambui, MG.

Maternal influence also contributed greatly to the construction of João Carlos^3^. Nicia was seemingly small in nature, yet she commanded the dynamics of the family with loving firmness, instilling sophisticated education and proper etiquette in the children. Modern in her time, she drove both cars and motorcycles. João Carlos was not short of examples of daring, inherited from both his father and mother. Her children listened to classical music and serenades, and read classic literature, of which João Carlos knew passages by heart, like the delusions and blunders of Don Quixote, which he would tell and double over with laughter, making us laugh along with him!

João Carlos was a great clinician, capable of identifying cardiac alterations without the use of instruments, simply by “listening” to the patient’s pulse^4^! For his doctoral thesis, he performed a retrospective study of Chagas disease in Bambui, where patients were initially diagnosed in the acute phase by his father in the 1940s and subsequently followed clinically by João Carlos. In this study, he made important observations about the occurrence of triatomines in the municipality, a focus of great importance in his life. In Bambui, he assembled what can be considered the first experience of municipalizing a vector control program, through an official partnership between the National Institute of Rural Endemic Diseases (incorporated into Fiocruz in 1970), the Superintendency of Campaigns of Public Health, and the local government of Bambui^5^. The proposal itself was distinguished by its approach to community participation, where residents were not limited to merely notifying the presence of kissing bug foci. Participation was to be obtained through a broad educational process^1^, strengthening citizens’ rights with strong political and ideological content, aligned with the transformative ideas of Paulo Freire^6^. This entomological surveillance program was subsequently expanded to 280 municipalities in the state of Minas Gerais^7^. It was later adopted as the model for surveillance across all of Brazil, and eventually, for other countries in Latin America.

Fearless, João Carlos played a fundamental role in exposing how people affected by Chagas disease lived, delving into the reality of rural life, from their housing to the uncertainty of living hand-to-mouth, collaborating so that the disease was perceived from a social perspective, mobilizing politics, and, emotionally, the very different people in the fight for the control of Chagas disease^1^. When urbanization brought to the cities people infected with *T. cruzi*, the etiological agent of Chagas disease, at a time when the current structure of the national health service (a Sistema Único de Saúde, SUS) did not exist, João Carlos created a system for the control of the transfusional transmission of Chagas disease, by subjecting blood donors every six months to tests, that resulted in a “donor card.” To this end, it was necessary to establish a dialogue with the Secretary of State for Health and a workflow to create a serology laboratory in the Fundação Ezequiel Dias (FUNED, the foundation that bears the name of the paternal grandmother of João Carlos, Ezequiel Dias, which today is the national reference for the serological diagnosis of Chagas disease), and an outpatient clinic in the Faculty of Medicine to assist seropositive donors. The system of donor cards included serology for Chagas disease, hepatitis, and syphilis. AIDS had not yet appeared, and with AIDS, the priority for the control of blood banks changed. João Carlos, in his experience and insight, was capable of panoramic analysis, identifying the problem, the chain of actions to solve it, promoting rapprochement and agreements, and creating new spaces for the care of people affected by Chagas disease.

João Carlos was then summoned to assume national responsibility for the “Program of Control of Chagas Disease” in Brasília^8^. At this point, the phase of attack against triatomines was well advanced, but it was necessary to ensure the continuity of the program, as dengue had entered the country and required funding. João Carlos’ performance in the Ministry of Health was fundamental for maintaining the program and advancing the implementation of epidemiological surveillance against Chagas disease.

In the 1990s, the spectacular results of the “Program of Control of Chagas Disease” became evident, marked by a reduction in intradomiciliary infestation by triatomines and a decline in the prevalence of infection in children. Epidemiological analyses indicated that *Triatoma infestans* is the principal vector species^9^. During a conversation in the backyard of Chris Schofield’s house, situated on the border between France and Switzerland, the two friends dared to dream of a collaborative effort to eliminate this triatomine from the countries where it occurred. Thus, “Dream 1” was born-the Intergovernmental Initiative for the Elimination of the Transmission of *T. cruzi* by *Triatoma infestans* in the Southern Cone Countries. Operationally led by the Pan American Health Organization, it played a fundamental role in consolidating the Brazilian program and advancing disease control in various other countries. Inspired by this initiative, “Dream 2” was established, successfully eliminating *Rhodnius prolixus* from Central American countries^10^.

Through intense engagement with the Sociedade Brasileira de Medina Tropical (SBMT), alongside their masters Aluizio Prata, Chapadeiro, and others, they created the “Meeting on Applied Research in Chagas Disease,” a platform for integrating research and service, always focused on solving problems and discovering new paths. Now preparing for its 41^st^ event, this meeting has become a permanent fixture in the SBMT congress program.

João Carlos was also president of the National Foundation of Health (Fundação Nacional de Saúde, FUNASA), and upon returning to Minas Gerais, also assumed coordination for the state of FUNASA and later became the Superintendency of FUNED^9^. During this period, João Carlos created essential services for controlling Chagas disease through diverse and innovative ways.

Returning to Fiocruz, he continued his research and integrated into a postgraduate course, where he coordinated a major discipline (always captivating us with his classes!). He mentored numerous students in scientific initiation, master's, doctoral, and post-doctoral programs, and coordinated the Ethics Committee for Research Involving Human. As one of the most productive researchers of Fiocruz, he also provided advice and consulting services to a wide range of national and international institutions. At the WHO, he served as a consultant for defining priority topics for investment in research on neglected diseases (Strategic and Technical Advisory Group on Neglected Tropical Diseases, WHO, Geneva)^9^. Even after his mandatory retirement, he arrived early at the lab, continuing at the same pace as the Emeritus Researcher of Fiocruz, a title he received on October 2, 2009. In 2006, João Carlos assumed the seat held by his father, Emmanuel, as a patron of the Minas Gerais Medical Association^9^. His conduct consistently contributed to strengthening the SUS, helping to consolidate the institutional mission of Fiocruz.

With approximately 250 papers published in top scientific journals, as well as books (13), book chapters (88), and technical publications (73), João Carlos has received countless decorations and prestigious awards.

- Decoration of the National Order of Scientific Merit Conferred by the President of the Republic.
- The Medal of Honor of the Inconfidência of the Government of the State of Minas Gerais,
- Carlos Chagas Medal of the Order of Medical and Scientific Merit from the Ministry of Health of Brazil.
- Many tributes from various Brazilian municipalities, such as Bambui, where he was an honorary citizen, Oliveira, and many other cities, such as Camiri, Salta, Tupiza, Tarija, Sucre, Luribay (Bolívia), Codba (Chile), Artigas (Uruguay), and Salta (Argentina).

Happy are fortunate to have had the opportunity to live alongside João Carlos! My teacher and mentor, following in his footsteps, I am strengthened in the pursuit of research that is committed to reality-transformative, capable of promoting citizenship and the dignity of people.

It is a great honor for the Sociedade Brasileira de Medicina Tropical to have João Carlos Pinto Dias as its founding member. We are grateful for his rich and tireless dedication to his remarkable legacy in both national and international sciences. We are all students of João Carlo.

We are grateful to Rosinha; to their children Mariana, Ruth, Lucia, and Marcos; to his brother Ezequiel; to his eight grandchildren and great-grandchild; to João Carlos; for forming this beautiful family; and for teaching us to overcome the strength of spirit and love the departure of João Carlos.

Thank you, João Carlos! Your memory will forever be that of an exceptional man-a symbol of affection, joy, and gratitude.
