# ERRATUM: Dr. João Carlos Pinto Dias, master and mentor! (★1948 †2021)

**DOI:** 10.1590/0037-8682-e0076b-2026

**Published:** 2026-08-03

**Revista da Sociedade Brasileira de Medicina Tropical/Journal of the Brazilian Society of Tropical Medicine**

Title: João Carlos Pinto Dias, master and mentor! (★1948 †2021) 

 Diotaiuti LG. Dr. João Carlos Pinto Dias, master and mentor! (★1948 †2021). Rev Soc Bras Med Trop. 2026 Apr 17;59:e00762026. doi: 10.1590/0037-8682-0076-2026. PMID: 42018914; PMCID: PMC13089445. 

 Dr. João Carlos Pinto Dias, master and mentor! (★1948 †2021) 

**Is correct**

 Dr. João Carlos Pinto Dias, master and mentor! (★1938 †2025) 

